# Change in Micro-Morphology and Micro-Mechanical Properties of Thermally Modified Moso Bamboo

**DOI:** 10.3390/polym14030646

**Published:** 2022-02-08

**Authors:** Tiancheng Yuan, Yaqian Huang, Tao Zhang, Xinzhou Wang, Yanjun Li

**Affiliations:** 1 Jiangsu Co-Innovation Center of Efficient Processing and Utilization of Forest Resources, Nanjing Forestry University, Nanjing 210037, China; ytc_njfu@163.com (T.Y.); huangyaqian1997@163.com (Y.H.); zt@njfu.edu.cn (T.Z.); xzwang@njfu.edu.cn (X.W.); 2 Bamboo Engineering and Technology Research Center, State Forestry and Grassland Administration, Nanjing 210037, China

**Keywords:** bamboo, saturated steam, nanoindentation, micro-mechanical properties

## Abstract

In recent years, saturated steam heat treatment has been considered as an environmentally friendly and cost-effective modification method compared with traditional heat treatment media. In this study, bamboo was treated by saturated steam, and the change in chemical composition, cellulose crystallinity index, micro-morphology, and micromechanical properties were analyzed by a wet chemistry method, Fourier transform infrared (FTIR), scanning electron microscopy (SEM), X-ray diffraction (XRD), nanoindentation, and so on. Results illustrated that the parenchyma cell walls were distorted due to the decomposition of hemicellulose and cellulose in bamboo samples. As expected, the hemicellulose and cellulose content decreased, whereas the lignin content increased significantly. In addition, the cellulose crystallinity index and thus the micromechanical properties of bamboo cell walls increased. For example, the hardness increased from 0.69 GPa to 0.84 GPa owing to the enhanced crystallinity index and lignin content.

## 1. Introduction

Due to global climate change, there is an urgent need to find a woody material that can replace wood and thus reduce the deforestation of native trees and the loss of forest resources [1,2]. It is necessary to find alternative woody materials which are low cost, have excellent mechanical properties, and are easy to harvest, versatile, and renewable, to solve the above problem [3,4,5,6,7]. In recent years, bamboo has gained more attention due to its abundance, sustainability, excellent physical properties, and ease of harvest. The basic composition of bamboo is hemicellulose, lignin, cellulose, tannins, waxes, resins, and so on. When grown in an outdoor setting, bamboo is prone to being attacked by fungi and insects due to its abundance of nutrients. Several modification methods have been applied to decrease the hygroscopicity and increase the durability of bamboo and bamboo-based products, such as chemical modification, acetylation, and thermal modification [8,9,10,11]. Thermal modification is a useful approach for increasing mechanical properties and dimensional stability [12,13].

When the heat energy is transferred, the key factor in the heat treatment process is the heat treatment medium. Conventional heat treatment media are usually oil, hot water, hot air, and heating is conducted at a high frequency, which does not provide a mild heat treatment condition for bamboo and wood. The surface of the bamboo or wood is heavily cracked after conventional heat treatment due to the loss of moisture. However, through visits to a number of companies, we have found that saturated steam is now a common heat treatment medium in factories. Saturated steam heat treatment has distinct superiority because it is eco-friendly, cost-effective, a simple operation, and does not require chemical agents among the several modification methods. In addition, the presence of water in the saturated steam modification process can accelerate the transfer of heat. The combination of pressure and steam promotes the decomposition of the hemicellulose in bamboo and bamboo-based products. Decomposition of hemicellulose begins when the treatment temperature reaches 120 °C, but significant decomposition with positive influence occurs above 160 °C. Unfortunately, some physical properties of bamboo are decreased due to the disruption of hydrogen bonds when the treatment temperature is above 180 °C. Changes in chemical composition, density, EMC, and mechanical properties of thermally modified wood have been intensely investigated at the macro scale [14,15,16]. In addition, micro-morphology and nanomechanical properties of thermally modified bamboo have been less intensely analyzed at the nano-scale. The nanoindentation (NI) technique is a successful approach for measuring the micro-mechanical properties of woody materials on the nano-scale. However, literature on how saturated steam heat treatment affects the creep ratio of bamboo at the nano-scale has not been reported yet.

The objective of this paper is to analyze the effect of saturated steam treatment temperature on the micro-morphology and micro-mechanical properties of thermally modified Moso bamboo cell walls. Aiming toward a deep and comprehensive understanding, changes in micro-morphology, micro-mechanical properties, crystallinity index, and chemical composition were investigated via scanning electron microscope (SEM), X-ray diffraction (XRD), Fourier transform infrared (FTIR), and nanoindentation (NI).

## 2. Materials and Methods

### 2.1. Materials

Six-year-old Moso bamboo (Phyllostachys edulis) without obvious defects was collected from Gaoan City, JiangXi Province, China. Then, the bamboo was processed into 1050 mm long bamboo culms. Finally, the bamboo culms were transferred to saturated steam equipment for thermal modification at different temperatures (160 °C, 170 °C, 180 °C) and the same duration (15 min). After saturated steam heat treatment, the untreated bamboo samples and treated bamboo samples were obtained. A0, A1, A2, and A3 represent the treated bamboo, treated at 160 °C, 170 °C, 180 °C, respectively. Bamboo culms were not dried after the treatment. The moisture content of the bamboo after processing was between 80 and 100%.

### 2.2. X-ray Diffraction (XRD)

The above-mentioned bamboo fiber powders were sieved through a 0.075 mm (200 mesh) sieve, in an attempt to obtain bamboo fiber powders with a particle size of less than 0.075 mm. The bamboo fiber bundles were tested with an X-ray diffractometer (XRD) (Ultima IV, Rigaku Corporation, Japan): target type Cu, radiation Cu-Kα, voltage 40 kV, and current 25 mA; continuous scanning, scanning step 0.02°, scanning speed 5°-min^−1^. The scanning range was 2θ = 5° − 40°. The crystallinity degree of cellulose can be calculated as follows:

CrI = (I_002_ − I_am_)/I_002_ × 100% (1)

where CrI represents the crystallinity index, Iam represents the minimum intensity of the amorphous material, and I002 represents the maximum intensity of the diffraction.

### 2.3. The Measurement of Oven-Dried Density and Thickness Shrinkage 

Firstly, bamboo samples with dimensions of 10 mm × 10 mm × thickness (mm) were prepared from treated bamboo culms. The prepared bamboo blocks were then tested for to obtain their oven-dried density. Before putting the bamboo blocks into the oven (103 °C), we recorded the initial mass and volume. The change in mass was recorded every two hours and when the weight remained the same, we considered the moisture content of the bamboo samples to be 0%. The oven-dried density can be calculated via the mentioned method. Both ends of the bamboo culms were marked with 6 separate test points. A micrometer screw was used for measuring the thickness of the bamboo culms before and after saturated steam heat treatment so that the average thickness shrinkage could be obtained.

### 2.4. Fourier Transform Infrared (FTIR)

Treated bamboo powder of 200 mg was tested with a range of 500–4000 cm^−1^ on a VERTEX 80 V FTIR spectrometer (Bruker Corporation, Karlsruhe, Germany). Fourier transform infrared (FTIR) was applied for analyzing the changes of chemical groups in bamboo samples after treatment. 

### 2.5. Measurement of Chemical Compositions in Bamboo Specimens

The treated bamboo samples were ground and screened into powders with size 40–80 mesh (0.075 mm–0.178 mm) for chemical composition analysis. The main chemical composition (cellulose, hemicellulose, and lignin) of the samples was tested according to NREL’S LAPS [17,18,19,20].

### 2.6. Nanoindentation (NI)

**Figure 1:**
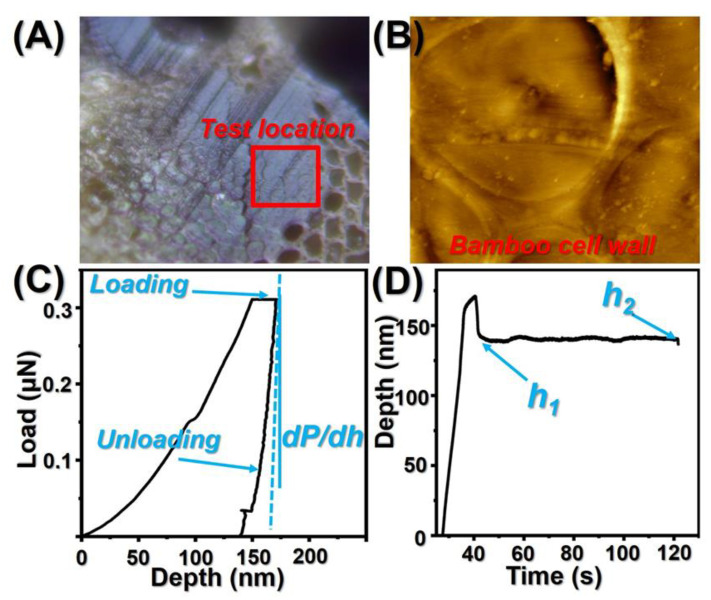
(**A**) Test location of treated bamboo cell walls; (**B**) AFM images of tested bamboo cell walls during the nanoindentation test; (**C**) nanoindentation load-depth curve of the bamboo sample; (**D**) The nanoindentation depth–time curve of bamboo specimens.

The specimens left for cross-sectional morphological analysis were adopted as test samples, and the surface of these samples was polished with an ultra-thin slicer equipped with a diamond knife, in order to obtain areas of fibers with a surface roughness of less than 10 nm. The cell areas were imaged with an AFM built into a nanoindenter (Ti800, Hysitron Inc., Irvine, CA, USA), as shown in Figure 1. The test location was chosen at the secondary wall layer of the cell wall and six cells were randomly selected on each specimen for testing. The test was performed in a three-stage constant rate loading and unloading mode (loading/holding/unloading for 5 s) with a maximum load of 400 μN. The test location was rescanned at the end of the test to obtain indentation images and 30 valid indentation points were selected. The loading–displacement curves of these valid points were statistically analyzed according to the Oliver and Pharr method [21,22]. The modulus of elasticity of bamboo cell walls can be calculated as below:

(2) $$H=\frac{P_{max}}{A}$$

where *P_max_* is the peak load, and *A* is the projected contact space of the indents at peak load. The hardness of different treated bamboo specimens can be calculated as follows:

(3) $$Er=\frac{\sqrt{\pi }}{2\beta }\frac{S}{\sqrt{A}}$$

where *Er* is the combined elastic modulus of both the sample and indenter; *S* is initial unloading stiffness; and *β* is a correction factor correlated to indenter geometry (*β* = 1.034).

Samples were loaded within 5 s, holding time was 5 s, and samples were unloaded within 5 s. The peak load (400 μN) was applied on all indents. Creep behavior can be calculated as follows:

(4) $$C_{IT}\left(\%\right)=\frac{h_{2}-h_{1}}{h_{1}}\times 100$$

where *h*2 and *h*1 represent the final and first penetration depth of the segment, respectively.

### 2.7. Statistical Analysis

Tukey’s tests were used to distinguish the differences between the untreated bamboo samples and treated groups via statistical analysis software SPSS. In addition, 12 replicates were used to measure means for oven-dried density, thickness shrinkage, modulus of elasticity, and hardness. Three replicates were used for cellulose crystallinity degree, main chemical component, and chemical groups in bamboo samples. The images in this article were drawn using Origin v9.0. Different capital letters represent the significant differences between heat treatment groups (*p* < 0.05).

## 3. Results 

### 3.1. SEM Analysis

**Figure 2:**
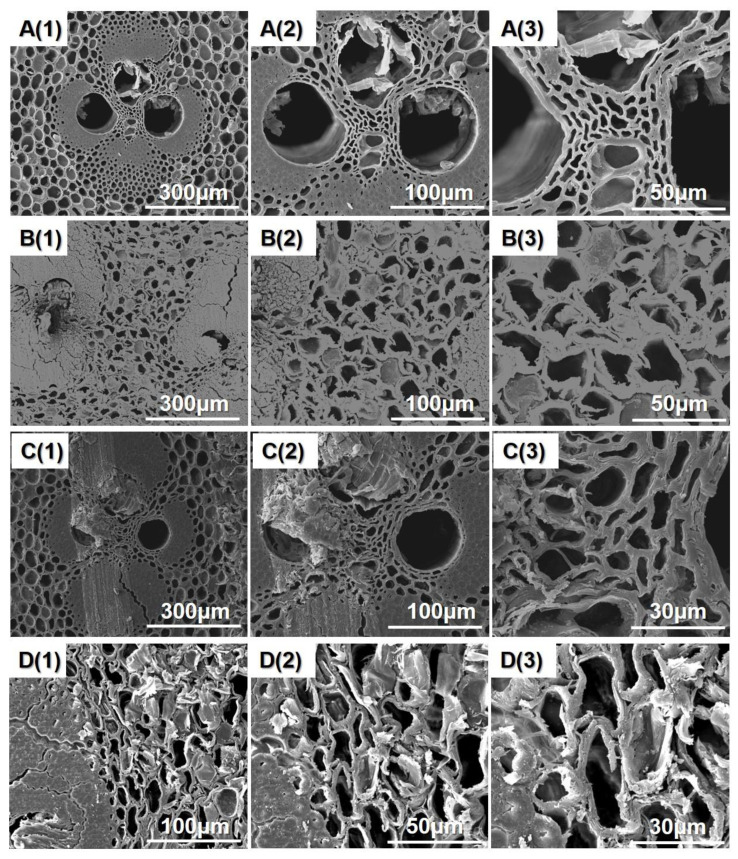
The cross-section images of differently treated bamboo samples: **A(1–3)**: A0; **B(1–3)**: A1; **C(1–3)**: A2; **D(1–3)**: A3.

Figure 2 presents a cross-section of bamboo cell walls at different size scales. For untreated bamboo specimens (Figure 2A(1–3)), the cross-sectional images exhibit intact vascular bundles and parenchyma cells. Scanning electron microscopy observation revealed the deformations of bamboo cell walls after thermal modification. The degradation of chemical components in bamboo cell walls may result in the deformation of bamboo cell walls during the thermal modification process [23]. The change of chemical composition needs to be further explored.

### 3.2. Density and Thickness Shrinkage

**Figure 3:**
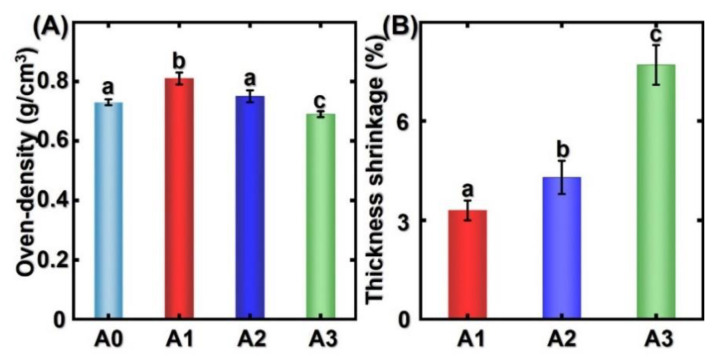
The (**A**) oven-density and (**B**) thickness shrinkage of different bamboo specimens. Different capital letters represent the significant differences between heat treatment groups (*p* < 0.05). The error bar in the picture represents the standard deviation.

Figure 3 presents the density and thickness shrinkage of thermally modified bamboo samples. As shown in Figure 3A, the oven-dried density of bamboo samples increased and then decreased with the increment of treatment temperature. For A1, the bamboo cell walls shrank because of the pressure and the plasticization of materials due to the saturated steam. After the thermal modification, the bamboo cells compacted so that the density of the bamboo increased in comparison with the untreated sample. However, the density of the bamboo showed a downward tendency when the treatment temperature was enhanced. This can be attributed to the hydrolysis and pyrolysis process during the thermal modification, and the chemical composition may change significantly at 180 °C. Results from statistical analysis showed that with the increasing treatment temperature, the shrinkage in the thickness of the bamboo samples increased statistically significantly. This was due to the decomposition of chemical compositions in bamboo cell walls, resulting in the decrement of thermally modified bamboo density [24,25].

### 3.3. Chemical Composition, XRD, and FTIR Analysis

**Figure 4:**
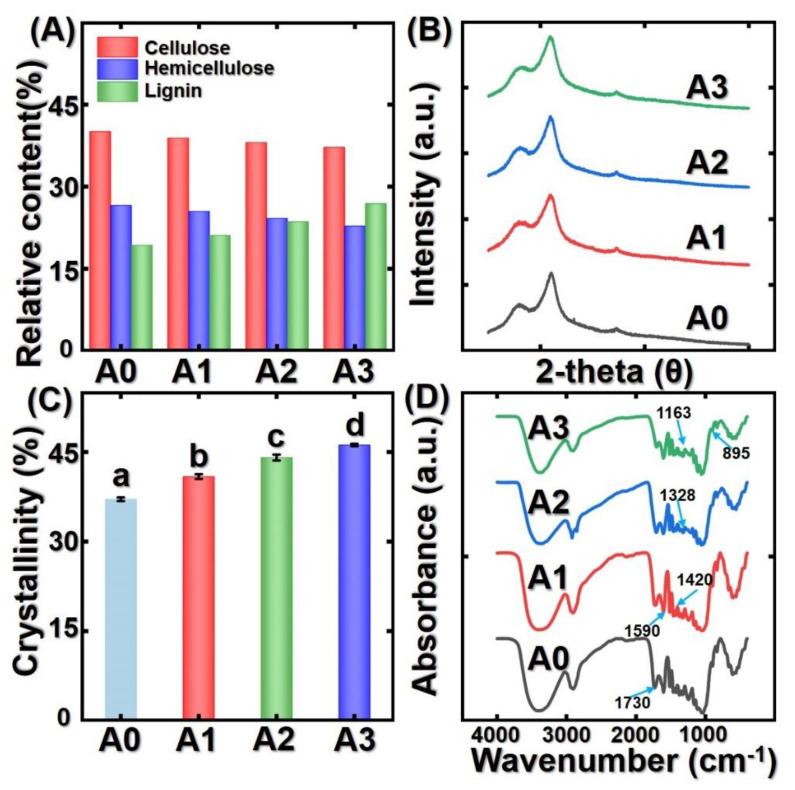
Multi-scale characterization of the change in untreated bamboo samples and treated bamboo samples: (**A**) relative content of chemical composition; (**B**) XRD curves of different bamboo samples; (**C**) crystallinity index of different bamboo samples; (**D**) FTIR curves of different bamboo samples. Different capital letters represent the significant differences between heat treatment groups (*p* < 0.05). The error bar in the picture represents the standard deviation.

As shown in Figure 4A, the relative contents of cellulose, hemicellulose, and lignin in untreated bamboo specimens were 40.1% (39.90 mg/92.27 mg), 26.6% (24.54 mg/92.27 mg), and 19.3% (17.80 mg/92.27 mg), respectively. It can be seen from Figure 4A that the saturated steam heat treatment has a significant effect on the relative content of cellulose, hemicellulose, and lignin in bamboo samples. The relative content of hemicellulose and cellulose decreased in comparison with the control. In addition, the hemicellulose and cellulose decreased continuously with the increase in treatment temperature. For example, the hemicellulose content decreased by 14.3% after treatment with the temperature at 180 °C. The volatilization of small molecular decomposition products is the main reason for the decrement of cellulose content. The hemicellulose is easily decreased because of its easy pyrolysis and poor thermal stability. Furthermore, xylose and arabinose are two key components in hemicellulose. However, xylose is easy to hydrolyze and pyrolyze due to its unstable branched structure. Fortunately, hemicellulose easily absorbs moisture in comparison with lignin and cellulose. Therefore, the decomposition of hemicellulose can enhance dimensional stability. Different from the decrement of hemicellulose and cellulose content, the lignin content increased as a function of treatment temperature. For example, the relative content of lignin increased significantly from 19.3% to 26.9%, which is co.19.3% enhancement. It can be attributed to the lignin condensation reaction with the by-products formed by the degradation of hemicellulose during the saturated steam heat treatment. Moreover, the degradation of cellulose and hemicellulose content is another reason for relative lignin content increment [26,27,28,29,30].

XRD curves of the different thermal modified bamboo samples are shown in Figure 4B. As shown in Figure 4B, the XRD curves of the untreated bamboo and saturated steam treated bamboo are basically the same, and showed two main diffraction peaks. The relative crystallinity index of bamboo specimens can be calculated by Equation (1). As expected, the relative degree crystallinity index of untreated bamboo (37.5%) is lower than that of treated bamboo samples. The crystallinity index of bamboo samples increased as a function of treatment temperature (Figure 4C). It can be analyzed by the decrement of the amorphous material in hemicellulose and cellulose. Additionally, the several acids formed by the thermal modification can diffuse in the para-crystalline part of cellulose, resulting in its decomposition, causing the crystallinity index of bamboo samples to increase further. Meanwhile, the acids can catalyze the decomposition of hemicellulose resulting in the pyrolysis of the amorphous part of the bamboo which can increase the cellulose crystallinity degree [31,32,33,34,35]. 

Figure 4D presents the FTIR curves of different bamboo samples. It can be seen from Figure 4D that the absorbance band is at 3400 cm^−1^ which corresponds to hydroxyl. The decrement of this absorbance band represents the number of hydroxyl groups oxidized in the hemicellulose. The aldehyde, ketone, and carboxyl groups are formed by the free hydroxyl polymerization. Thus, with the hemicellulose and cellulose decreased, the hydroxyl group decreased. The absorbance bands at 1730 cm^−1^ and 1590 cm^−1^ correspond to the non-conjugated C=O stretching vibrations and aromatic skeletal vibrations (Figure 4D). The intensity of this absorbance peak decreased, confirming the decomposition of hemicellulose. Additionally, the intensity of the absorbance peak at 1420 cm^−1^ changed slightly, illustrating the loss of C=O groups in aromatic skeletal vibration. According to previous literature [31,32,33], the absorbance peak at 1420 cm^−1^ is to be considered unchanged during the thermal modification process. The increment of the spectral region between 1328 cm^−1^ corresponded to a C-O stretching vibration due to the increment of lignin content. The absorption peak at 1163 cm^−1^ (C-O-C stretching vibrations in the amorphous part of cellulose) and 895 cm^−1^ (C-H in cellulose). The intensity of these two peaks decreased due to the degradation of amorphous cellulose. The analysis of the change in chemical composition groups confirmed the decomposition of hemicellulose and cellulose.

### 3.4. Micro-Mechanical Properties of Bamboo Cell Walls

**Figure 5:**
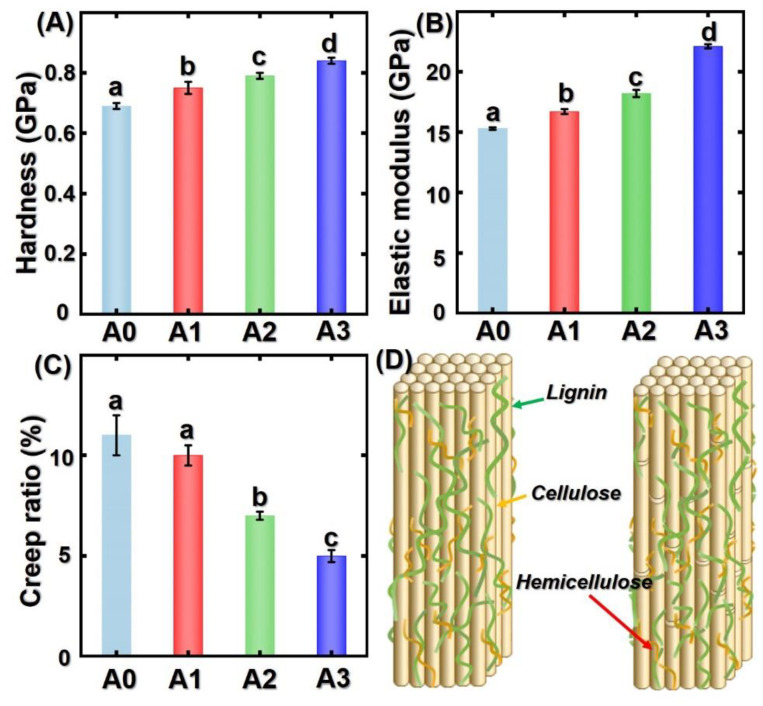
Micromechanical properties of bamboo specimens: (**A**) hardness; (**B**) elastic modulus; (**C**) creep ratio; (**D**) schematic diagram of thermal modification process. Different capital letters represent the significant differences between heat treatment groups (*p* < 0.05). The error bar in the picture represents the standard deviation.

In Figure 5, the average hardness and elastic modulus of untreated bamboo cell walls were 0.69 GPa and 15.3 GPa, respectively. Treatment temperature contributed positively to the micromechanical properties of treated bamboo specimens. For instance, the average hardness increased significantly from 0.69 GPa to 0.84 GPa after saturated steam heat treatment. The micromechanical mechanics of bamboo cell walls were usually affected by cellulose crystallinity degree, chemical composition, moisture content, density, and lignin content. In this study, the modulus of elasticity showed the same increasing tendency. It can be attributed to the change of matrix and arrangement of cellulose microfibrils in the bamboo cell walls [36,37]. Nanoindentation is a useful technology for measuring the polymers’ hardness and modulus of elasticity. Meanwhile, bamboo specimens exhibit creep behavior during the process of the load–unload indentation test. As shown in Figure 5C, the higher the treatment temperature, the smaller the creep ratio of all bamboo specimens. In other words, thermal modification temperature positively contributed to decreasing creep ratio. As we know, hemicellulose is an important component of cell wall plasticity. After saturated steam heat treatment, the relative hemicellulose content decreased, as did the stiffness and rigidness of bamboo cell walls. Thus, the resistance to creep of treated bamboo cell walls was enhanced due to the degradation of polymers in cell walls. In addition, the recondensation of lignin and increased cellulose crystallinity index may also have contributed to this observation [38,39,40,41].

### 3.5. Proposed Mechanism

In Figure 5D, we have illustrated the thermal modification mechanism of the changes in the main composition of bamboo after saturated steam heat treatment. During the thermal modification process, the acetic acid formed from the acetyl groups accelerated the decomposition of the hemicellulose in bamboo cell walls. For lignin, the β-o−4 bonds were cleaved, resulting in the loosening of the intermolecular linkages in lignin. In addition, the ferulic acid has also been broken, which plays an important role in connecting hemicellulose and cellulose. Therefore, the intact structure of the cell walls decomposed. On the macroscale, the distorted parenchymal cell and decreased water-absorbing ability confirmed this observation. Due to the decrement of hemicellulose in cell walls, the number of O-H bonds decreased at the same time, and thus the dimensional stability of bamboo samples increased. 

## 4. Conclusions

In this study, saturated steam heat treatment enhanced the dimensional stability of bamboo samples due to the decomposition of hemicellulose. Results showed that parenchyma cells deformed due to the degradation of chemical composition in bamboo cell walls. The lignin content showed an increasing tendency whereas the hemicellulose and cellulose decreased with increasing treatment temperature. However, the modulus of elasticity and hardness of thermally modified bamboo cell walls increased by nearly 21% and 35%, respectively. Reduced hemicellulose resulted in a decreased creep ratio. Likewise, the resistance to creep of bamboo cell walls increased due to the increased crystallinity index. Furthermore, these results can help the reader to deeply understand the change in micro-morphology and micro-mechanical properties of bamboo after saturated steam heat treatment.
